# Stroke risk among patients with chronic obstructive pulmonary disease: A systematic review and meta-analysis

**DOI:** 10.6061/clinics/2018/e177

**Published:** 2018-04-21

**Authors:** Yu Ree Kim, In Cheol Hwang, Yong Joo Lee, Eun Bee Ham, Dong Kyun Park, Sewan Kim

**Affiliations:** I Department Family Medicine, Gachon University Gil Medical Center, Incheon, Republic of Korea; II Department of Family Medicine, Catholic University Seoul St. Mary’s Hospital, Seoul, Republic of Korea; III Department of Internal Medicine, Gachon University Gil Medical Center, Incheon, Republic of Korea; IV Shingil Yonsei Clinic, Seoul, Republic of Korea

**Keywords:** Cerebrovascular Disease, Chronic Obstructive Pulmonary Disease, Meta-Analysis, Stroke

## Abstract

Increased stroke risk among chronic obstructive pulmonary disease patients has not yet been established. In this study, we conducted a systematic review and meta-analysis to assess stroke risk among chronic obstructive pulmonary disease patients. PubMed, EMBASE, and the Cochrane Library were systematically searched from database inception until December 31, 2016 to identify longitudinal observational studies that investigated the association between chronic obstructive pulmonary disease and stroke. Stroke risk was quantified by overall and subgroup analyses, and a pooled hazard ratio was calculated. Study quality was evaluated using the Newcastle-Ottawa Scale. Publication bias was assessed using Begg’s rank correlation test. Eight studies met the inclusion criteria. In a random-effects model, significantly increased stroke risk was observed among chronic obstructive pulmonary disease patients (hazard ratio, 1.30; 95% confidence interval, 1.18-1.43). In subgroup analyses stratified by stroke subtype, study quality, and adjustment by socioeconomic status, the association between increased stroke risk and chronic obstructive pulmonary disease patients was robust. Statistically significant publication bias was not detected. In summary, chronic obstructive pulmonary disease was found to be associated with increased stroke risk. Additional prospective studies are required to elucidate the mechanisms underlying the increase in stroke risk and identify effective preventive interventions.

## INTRODUCTION

Chronic obstructive pulmonary disease (COPD) is characterized by persistent and usually progressive airflow restriction 1. COPD may limit activity, compromise quality of life, and result in disability. As such, this disorder presents a major, incurable population health burden 2. In addition, COPD patients may suffer from additional systemic manifestations of the disorder. Comorbid medical conditions further contribute to disease severity, functional impairment, reduced quality of life, and poor clinical outcomes. Cardiovascular diseases (CVDs), including ischemic heart disease, atrial fibrillation, and heart failure, and cerebrovascular diseases are the most prevalent and fatal comorbidities of COPD 3.

While the coexistence of COPD and CVD is well established, whether COPD increases the individual’s risk for all types of CVD has not been confirmed. Shared risk factors, persistent inflammation, and vulnerability to infection have been proposed as mechanisms by which COPD and CVD might be linked 4. While there is evidence suggesting that COPD increases the risk of heart disease 4,5, studies conducted to date on the risk of stroke among COPD patients have generated conflicting results. A recent review of the current evidence on stroke risk among COPD patients was limited by a dependence on cross-sectional data, which precluded a firm establishment of causality between COPD and stroke 4.

To further investigate the relationship between COPD and stroke, we performed the present meta-analysis of longitudinal studies and evaluated incident stroke in COPD patient cohorts. The primary aim of this study was to clarify whether COPD patients are at increased risk of stroke. Secondary aims were to assess whether the association between COPD and stroke varies according to the type of stroke or the core adjusted variables, including socioeconomic status (SES). A systematic review of the literature on the association between stroke and COPD was undertaken in order to provide a comprehensive perspective on disease mechanisms in COPD and to identify potential strategies for stroke prevention among COPD patients.

## METHODS

### Search strategy

Three databases (PubMed, EMBASE, and the Cochrane Library) were systematically searched from their inception to December 31, 2016. The following search terms were used to identify potential studies for inclusion: (COPD OR chronic obstructive airways disease OR chronic obstructive bronchial disease OR emphysema OR chronic bronchitis) and (stroke OR cerebrovascular disease OR cerebrovascular accident OR cerebral ischemia OR ischemic stroke OR transient ischemic accident OR hemorrhagic stroke). No search filters were applied. We also identified additional papers by reviewing the relevant articles listed in the references. This review followed the meta-analysis of observational studies in epidemiology (MOOSE) guidelines.

### Study selection and inclusion criteria

Primary studies investigating stroke occurrence among COPD patients were eligible for inclusion. Additional eligibility criteria were that each study had to report the risk of developing stroke (e.g., not stroke-related mortality) and provide measures of association (hazard ratio (HR), incidence ratio (IR), or odds ratio (OR)) and precision (confidence interval (CI)). To investigate causality, we further narrowed the inclusion criteria to include only observational studies using a longitudinal study design. In the case of multiple publications using the same study population, only the most recent, largest, or most relevant publication was selected for inclusion.

### Data extraction

Databases searches and data extraction were conducted by two independent investigators (YRK and YJL). Disagreements were resolved through consultation with a third author (ICH). The following information was extracted from each study: first author, year of publication, country, study setting, study design, measures of association with the corresponding 95% Cis, and core adjusted variables. Fully adjusted measures of association were extracted from the included studies. The Newcastle-Ottawa Scale, which is mainly based on potential confounding variables in the study populations, was used to assess methodological study quality.

### Statistical analysis

This investigation aimed to assess whether there is an increased risk of incident stroke among COPD patients compared to control subjects. Subgroup analyses were performed for stroke subtype (all types, ischemic stroke, or hemorrhagic stroke); study quality (low or high); and consideration of SES (unadjusted or adjusted). HRs (with 95% CIs) from the individual studies were pooled. Using well-established methods 6 for calculating the variance of log HR from each study, we converted the 95% CIs to their natural logarithmic value by dividing the range of the CI by 3.92. We used Higgins *I^2^* (0-100%) to assess the heterogeneity across studies. When substantial heterogeneity (*I^2^*≥50%) was observed, we used the DerSimonian and Laird random-effects model as previously described 7. Random-effects models attempt to generalize findings beyond the included studies by assuming that the selected studies are random samples from a larger population 8. We also assessed publication bias using Begg’s rank correlation test. Stata software (version 12.1, StataCorp, College Station, TX, USA) was used for all statistical analyses.

## RESULTS

**Figure 1:**
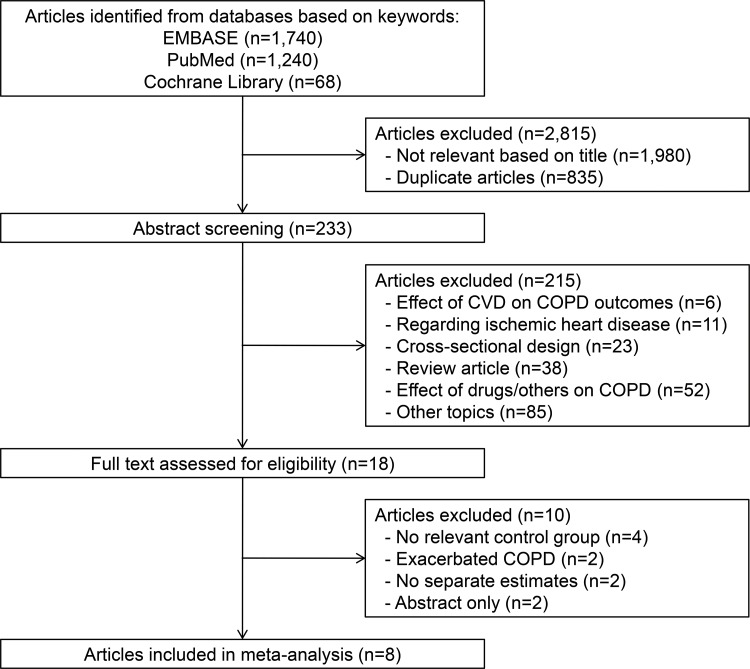
Flow diagram of the identification of relevant articles.

Figure 1 presents the flow diagram of the identification of relevant studies. From the 3,048 articles that were initially retrieved, 233 underwent abstract review, and 18 articles underwent full-text review. Ten articles were subsequently excluded for the following reasons: no relevant control group (n=4); no separate estimate of COPD association with stroke (n=4); or no estimates on exacerbated COPD (n=2). A total of eight studies were included in the final analysis.

Characteristics of the included studies are summarized in Table 1. Most studies were conducted in Western countries (n=7) and investigated newly developed stroke (n=6). Only one study utilized a prospective cohort design. The core adjusted variables were metabolic comorbidities (hypertension, type 2 diabetes, and hyperlipidemia); prior CVD (ischemic heart disease and stroke); lifestyle (cigarette smoking, alcohol consumption, and obesity); and SES. Five recently published studies were rated as high-quality studies.

**Figure 2:**
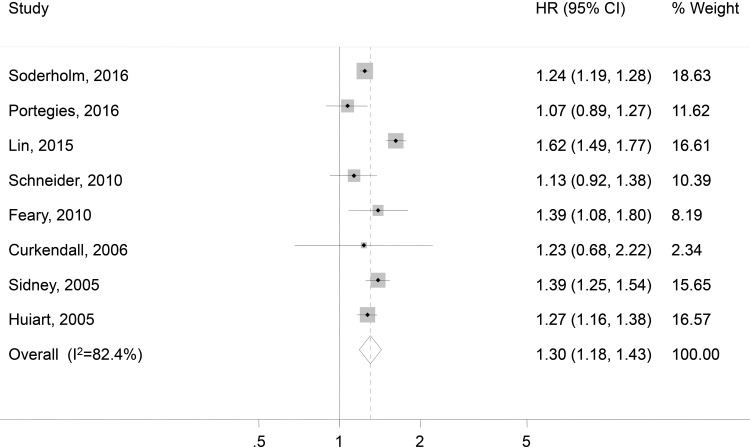
Forest plot showing the risk of stroke among COPD patients. HR, hazard ratio; and CI, confidence interval.

**Figure 3:**
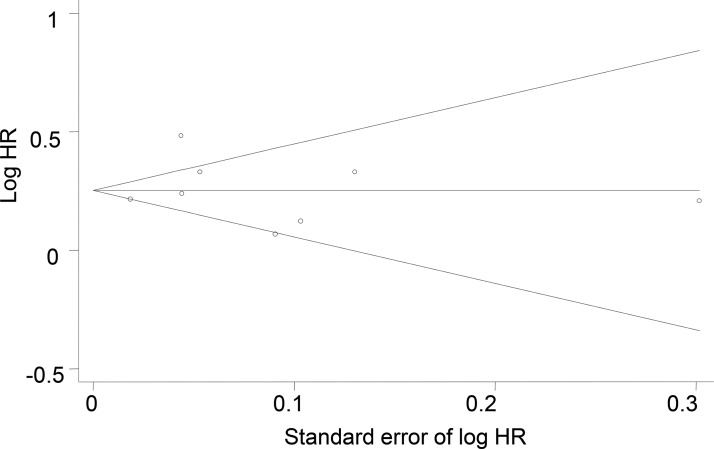
Begg’s funnel plot for incident stroke among the included studies (*p*=0.135). HR, hazard ratio.

Overall, a significantly increased risk of stroke was observed among COPD patients (HR, 1.30; 95% CI, 1.18-1.43; *p*<0.001; Figure 2). No single study unduly impacted the results. The association between COPD and stroke risk remained robust in subgroup analyses by stroke subtype, study quality, and adjustment for SES (Table 2). Interestingly, high-quality studies and studies that adjusted for SES variables tended to have smaller effect sizes. Funnel plot asymmetry among studies was not observed (*p*=0.135; Figure 3).

## DISCUSSION

As comorbidities may substantially influence the severity and prognosis of COPD, studies exploring the associations between specific comorbidities and COPD are urgently needed. Stroke carries a significant risk of morbidity and mortality and is one of the most common causes of death and severe disability. Increased risk of stroke among COPD patients will increase the burden on their caregivers, whom are prone to adverse mental health when looking after COPD or stroke patients 9,10. Stroke may also contribute to pulmonary dysfunction due to impaired cough and/or weakness of respiratory muscles, resulting in an increased propensity for pneumonia 11. Accordingly, incident stroke in COPD can accelerate and further increase mortality risk. In our pooled analysis, COPD patients were found to have a 30% increased risk of developing stroke compared with non-COPD subjects. The observed association between COPD and stroke risk remained robust in subgroup analyses by stroke subtype and after adjustment for core variables. These results suggest that stroke risk should be closely monitored and controlled in patients with COPD.

Our subgroup analyses suggest that the increased stroke risk among COPD patients is not merely due to confounding by common risk factors, such as smoking, metabolic comorbidities, or SES. There is no doubt that cigarette smoking is associated with an increased risk of CVD, including stroke, and indeed, smoking has been shown to be associated with preclinical brain changes on magnetic resonance imaging 12. However, in prior studies of COPD and stroke, risk estimates were reduced by only approximately 10% after adjusting for smoking 13,14. In addition, cerebral small vessel disease has been shown to be significantly more prevalent among smokers with COPD than smokers without COPD 15, suggesting an additional pathogenic effect of COPD beyond the harm from smoking itself. Socioeconomic inequalities may also potentially account for disparities among risk factors 16; however, in our analysis, SES did not critically impact the observed associations between COPD and increased stroke risk, which supports the existence of a COPD-specific effect.

Potential mechanisms by which COPD and stroke might be linked include increased systemic inflammation (for example, resulting in elevated interleukin-6 and C-reactive protein levels) and increased oxidative stress 17. COPD is a persistent airway inflammatory process and can potentially cause a continuous spillover of inflammatory mediators from the lungs into the blood stream 18. Many human studies have demonstrated increased serum levels of inflammatory and oxidative stress markers in COPD patients compared to controls 19,20. This dispersion of pro-inflammatory factors from the lungs may contribute to endothelial dysfunction and promote a pro-coagulative state, which may then lead to atherothrombosis, thereby providing a potential mechanistic link between COPD and the increased risk of cerebrovascular events 18. Furthermore, pro-inflammatory cytokines, including interleukin-6 and tumor necrosis factor alpha (TNF-α), were found to be elevated in depression 21. Depression is a common comorbidity of COPD 22 and stroke 23. This observation further strengthens the neuro-immunological link among smoking, cardiovascular risk factors, COPD and stroke 24. Furthermore, the incidence rate of stroke has been found to be significantly increased following acute COPD exacerbations 25,26. Such exacerbations represent times of intense oxidative stress and increased systemic inflammation 27. However, to date, no clinical data have suggested that suppression of inflammation prevents or improves CVD outcomes. For example, statin use was not beneficial for COPD patients, and use of an inhaled corticosteroid was not found to affect cardiac mortality 28,29.

Another mechanism by which COPD may specifically increase the risk of stroke is through hypoxemia. COPD patients, particularly those with more advanced disease, may experience hypoxemia, and subsequent hyperventilation contributes to vessel wall changes 30. Metabolic disturbances resulting from hyperventilation may also induce cardiac arrhythmias. Reduced lung function in COPD patients has been found to be inversely associated with incident atrial fibrillation, another condition that might predispose patients to stroke 31. COPD represents more than impaired lung function, but reduced lung capacity itself, regardless of the cause, has been associated with increased risk of atrial fibrillation 32,33, stroke 34,35 and CVD mortality 36.

In the current study, although the majority of stroke events among COPD patients were of the ischemic type, the association between COPD and the risk of hemorrhagic stroke was also robust. Levels of plasminogen activator inhibitor-1 (PAI-1), a major inhibitor of fibrinolysis, were found to be higher in COPD patients and may contribute to thrombosis and ischemic stroke among these patients 37. Plasminogen activators were found to be beneficial for patients with mild stroke 38. However, additional study is required to evaluate the efficacy and safety of plasminogen activators as a treatment for stroke among COPD patients. Some risk factors are shared across all stroke subtypes, while others are differentially related 39. Aging and smoking are known to disrupt the blood-brain barrier 40,41, which may contribute to the increased risk of hemorrhagic stroke among COPD patients. High blood pressure is also a major risk factor for hemorrhagic stroke 42, and accumulation of blood within the brain may rapidly lead to damage due to mechanical injury and increased pressure 43. Prevalent use of anticoagulant therapy for arrhythmias 3 in COPD patients may also partly explain the association between COPD and the increased risk of hemorrhagic stroke.

The present study has some limitations, mainly regarding study design, as it relied on data from studies using administrative healthcare databases. When using administrative healthcare data, there exists uncertainty regarding the diagnoses of COPD and stroke; healthcare databases usually do not contain records of lung function tests and brain imaging studies. Records from these sources also lack information on several important characteristics, including lifestyle factors such as smoking status, alcohol consumption, and physical and sedentary activity, thus preventing further stratified analyses. Variation among adjusted variables might partially account for the high heterogeneity found in the current meta-analysis. Another limitation of the included studies was that there was insufficient information on medication effects. COPD patients frequently use medications that may induce an acute CVD episode, including anticholinergic agents and sympathomimetic medications 44,45. COPD patients may also require treatment with macrolides and quinolone antibiotics, which are both associated with increased risk of adverse cardiovascular side effects 46. Finally, in this meta-analysis, there were not enough studies from non-Western countries, such as Asian studies. Further research is required to study the risk of stoke among Asian patients with COPD. At present, there are reports that Asian patients with COPD and stroke are more likely to report negative health and functional status 47. To further clarify the relationship between COPD and stroke risk, well-designed, prospective cohort studies with collaborating interventional trials are required. Further basic science studies are also required to explore whether targeting oxidative stress and lung and systemic inflammatory pathways may provide effective approaches for preventing vascular injury.

In conclusion, the results of this study suggest that COPD increases the risk of stroke, independent of other shared CVD risk factors. Current knowledge on the most effective approach for reducing stroke risk in COPD patients is still limited. For example, anticoagulant therapy is indicated only for COPD patients with atrial fibrillation 48. Primary care physicians should pay close attention to CVD risk, including stroke risk, from the time of COPD diagnosis, and where appropriate, they should work more closely with cardiologists and neurologists to enable optimal vascular care for patients with COPD.

## AUTHOR CONTRIBUTIONS

Kim YR, Hwang IC and Lee YJ contributed to the study conception and design, data acquisition and analysis, and manuscript drafting. All authors contributed to data interpretation and critical revisions of the manuscript. Hwang IC and Lee YJ contributed equally to this work.

## Figures and Tables

**Table 1:** Characteristics of studies reporting the risk of incident stroke among COPD patients.

First author, year	Country	Setting	Design	Outcomes	Stroke risk (95% CI)	Adjusted variables other than age and sex	NOS
Söderholm, 2016 (49)	Sweden	103,419 COPD/103,419 without COPD	Nested case-control	First stroke	All: 1.24 (1.19-1.28) IS: 1.20 (1.15-1.25) HS: 1.32 (1.20-1.45)^a^	Metabolic comorbidities^c^ Prior CVD Lifestyle (alcohol) SES Others comorbidities^d^	8
Portegies, 2016 (25)	Netherlands	1,566 COPD/11,549 without COPD	Prospective cohort	First stroke	All: 1.07 (0.89-1.27) IS: 1.10 (0.88-1.38) HS: 1.45 (0.86-2.45)	Metabolic comorbidities^c^ Prior CVD Lifestyle (BMI and smoking) Others (hs-CRP and cIMT)	9
Lin, 2015 (50)	Taiwan	10,413 COPD/41,652 without COPD	Nested case-control	First stroke	All: 1.62 (1.49-1.77) IS: 1.64 (1.49-1.82)^b^ HS: 1.18 (0.89-1.57)^b^	Metabolic comorbidities^c^ Prior CVD Lifestyle (BMI, smoking, and alcohol)	8
Schneider, 2010 (13)	UK	2,482 new COPD/6,116 without COPD	Nested case-control	First stroke	All: 1.13 (0.92-1.38)	Metabolic comorbidities^c^ Lifestyle (BMI and smoking)	8
Feary, 2010 (14)	UK	26,915 COPD/1,136,898 without COPD	Nested case-control	First stroke	All: 1.39 (1.08-1.80)^a^	Metabolic comorbidities^c^ Prior CVD Lifestyle (smoking) SES	8
Curkendall, 2006 (31)	Canada	11,493 COPD/22,986 without COPD	Nested case-control	Any stroke	All: 1.23 (0.68-2.22)	Metabolic comorbidities^c^ Prior CVD	7
Sidney, 2005 (51)	USA	36,931 COPD/44,137 without COPD	Nested case-control	First stroke	All: 1.39 (1.25-1.54)	Metabolic comorbidities^c^	7
Huiart, 2005 (52)	Canada	5,648 new COPD/general population	Nested case-control	Any stroke	All: 1.27 (1.16-1.38)	None	6

COPD, chronic obstructive pulmonary disease; CI, confidence interval; IS, ischemic stroke; HS, hemorrhagic stroke; CVD, cardiovascular disease; CRP, C-reactive protein; cIMT, carotid intima-media thickness; and NOS, Newcastle-Ottawa Scale.

a Recalculated.

b Not adjusted for lifestyle factors.

c Hypertension, diabetes, and hyperlipidemia.

d Including asthma, rheumatoid arthritis, lupus, and renal failure.

**Table 2:** Risk of developing stroke among patients with COPD.

	No. of studies	Summary HR (95% CI)	Heterogeneity, I^2^	Model
Stroke subtypes				
All stroke (13,14,25,31,49-52)	8	1.30 (1.18-1.43)	82.4%	Random-effects
Ischemic stroke (25,49,50)	3	1.31 (1.03-1.66)	94.0%	Random-effects
Hemorrhagic stroke (25,49,50)	3	1.31 (1.20-1.43)	0%	Fixed-effects
Study quality				
Low (31,51,52)	3	1.32 (1.23-1.41)	0%	Fixed-effects
High (13,14,25,49,50)	5	1.28 (1.10-1.50)	89.3%	Random-effects
SES variables				
Unadjusted (13,25,31,50-52)	6	1.30 (1.14-1.49)	82.5%	Random-effects
Adjusted (14,49)	2	1.24 (1.20-1.29)	0%	Fixed-effects

COPD, chronic obstructive pulmonary disease; SES, socioeconomic status; HR, hazard ratio; and CI, confidence interval.
